# Assessment of Mammalian Scavenger and Wild White-Tailed Deer Activity at White-Tailed Deer Farms

**DOI:** 10.3390/v17081024

**Published:** 2025-07-22

**Authors:** Alex R. Jack, Whitney C. Sansom, Tiffany M. Wolf, Lin Zhang, Michelle L. Schultze, Scott J. Wells, James D. Forester

**Affiliations:** 1 Department of Biology, Irving K. Barber Faculty of Science, University of British Columbia, Okanagan 1177 Research Road, Kelowna, BC V1V 1V7, Canada; 2 Department of Fisheries, Wildlife and Conservation Biology, University of Minnesota, 2003 Upper Buford Circle, Suite 135, St. Paul, MN 55108, USA; sanso025@umn.edu (W.C.S.); jdforest@umn.edu (J.D.F.); 3 Veterinary Population Medicine, AnSci/VetMed, University of Minnesota, 1988 Fitch Ave 495, St. Paul, MN 55108, USA; wolfx305@umn.edu; 4 Division of Biostatistics and Health, Data Science School of Public Health, University of Minnesota, Twin Cities, 2221 University Avenue SE, Suite 200, Minneapolis, MN 55414, USA; zhan4800@umn.edu; 5 136 Andrew Boss Laboratory, Center for Animal Health and Food Safety, College of Veterinary Medicine, University of Minnesota, 1354 Eckles Avenue, St. Paul, MN 55108, USA; schu3298@umn.edu; 6 College of Veterinary Medicine, University of Minnesota, 225 Veterinary Medical Center, 1365 Gortner Ave, St. Paul, MN 55108, USA; wells023@umn.edu

**Keywords:** chronic wasting disease, deer farms, camera traps, scavenging, white-tailed deer

## Abstract

White-tailed deer (*Odocoileus virginianus*) in the wild and on cervid farms have drawn the attention of state wildlife agencies and animal health agencies as Chronic Wasting Disease (CWD) has spread across North America. Deer farm regulations have been implemented to reduce direct contact between wild and farmed cervids; however, evidence suggests that indirect contact to infectious prions passed through the alimentary tracts of scavengers may be an important transmission pathway. The objective of this study was to characterize mammalian scavenger and wild deer activities associated with deer farms and link these activities with site-specific spatial covariates utilizing a network of camera traps, mounted to farm perimeter fences. We monitored each of 14 farms in Minnesota, Wisconsin, and Pennsylvania for two weeks during the summer, with a subset of farms also monitored in the winter and fall. Across all sites and seasons, we captured 749 observations of wildlife. In total, nine species were captured, with wild white-tailed deer accounting for over three quarters of observations. Despite the large number of wild deer observed, we found that interactions between wild and farmed deer at the fence line were infrequent (six direct contacts observed). In contrast, mammalian scavengers were frequently observed inside and outside of the fence. Supplementary cameras placed on deer feeders revealed higher observation rates of scavengers than those placed along fence lines, highlighting the potential for transmission of CWD through indirect contact via scavenger excreta. To evaluate associations between the number of observations of focal species with land-cover characteristics, two mixed-effects regression models were fitted, one model for scavengers and one for wild deer. Contrary to our hypothesis, landscape context did not have a strong impact on wildlife visitation. This suggests that farm location is less important than management practices, highlighting the need for future research into how farming practices impact rates of wildlife visitation onto cervid farms.

## 1. Introduction

Chronic wasting disease (CWD) is a fatal encephalopathy affecting at least seven species within the Cervidae family [1]. The disease is spreading extensively throughout North America [2]. CWD has been associated with population declines in white-tailed deer [3] (WTD), and such regional reductions could have ecological ripple effects. Additionally, concern over the disease has been linked to decreased hunter participation [4]. A decline in hunting may negatively impact not only wildlife agencies—which rely on revenue from hunting license sales [5]—but also the broader economy [6].

Chronic wasting disease is spread through a variety of pathways. It can be transmitted directly from host to host through typical cervid behaviors such as sparring, mating, and nose-to-nose contact [7]. However, indirect transmission can also occur. Infected hosts shed misfolded prions throughout the course of the disease via excreta, scraping, and carcass decomposition. These prions can bind to soil and plant tissues, where they remain infectious and can be taken up by susceptible cervids [8,9,10], and potentially by other wildlife species.

Although the risk of long-range transport of subclinical CWD+ deer has been reduced through awareness of direct transmission and the implementation of regulatory programs, relatively little research has addressed the potential for indirect transmission routes—especially the role of wildlife in introducing CWD to deer farms [2]. In a case–control study of 71 farmed deer herds across Pennsylvania, Minnesota, and Wisconsin, Schultze et al. [11] found that positive (case) herds were more likely than negative (control) herds to have received deer from farms later found to be CWD+, to be located near wild CWD+ deer populations, and to experience activity by mammalian scavengers (hereafter scavengers) along perimeter fences (hereafter fences).

Evidence is growing that infectious prions remain infectious after passing through the digestive tracts of various scavengers and predators [12,13,14,15,16]. Infectious prions have even been detected in fecal material from wild coyotes and cougars in CWD-endemic areas [17], raising concerns that these animals could facilitate environmental contamination and disease spread.

Given these findings, and the potential for scavengers to serve as indirect transmission vectors between wild WTD and farmed cervids, it is critical to better understand where and how often these interactions occur. Thus, the objective of this study was to quantitatively assess the frequency of wild deer and scavenger presence along the perimeter fences of deer farms, and to evaluate this activity in relation to spatial and temporal factors. We hypothesized that scavenger activity would be higher along forested fence segments, while wild deer activity would be greater in mixed-use landscapes containing rangeland, farmland, and forest. We also anticipated that interactions between wild and farmed deer would increase during the fall, and that scavenger activity would be more frequent at night. Additionally, we expected higher concentrations of scavenger activity near deer feeding sites (feeders), assuming omnivorous scavengers are attracted by food availability. By quantifying the frequency and timing of these interactions, this study aims to provide deer farm managers with actionable information to mitigate the risk of CWD transmission.

## 2. Materials and Methods

### 2.1. Study Area: CWD

In our study area (Pennsylvania, Wisconsin and Minnesota), CWD has been documented in wild populations of deer since the early 2000s. CWD was first detected in Pennsylvania in 2010 in the south-central portion of the state and has since increased to encompass 8 disease management zones across a large portion of the center of the state [18]. Since its initial detection in southwestern Wisconsin 2002, CWD has since spread to roughly 65% of the 72 counties in the state [19,20]. In Minnesota, the disease was first detected in wild deer in 2010 in the southeast [21]; however it has since been observed in wild populations in the central and northwestern portions of the state [22].

### 2.2. Description of Farms

Eligible properties were farms in MN, WI, and PA that were willing to allow cameras on their facilities. The properties varied in geographic shape, size, and the number of deer present (range: 7–200 individuals; Table 1). Several properties were hunting ranches (*n*= 2), which specialized in rearing trophy deer for hunting on-premises. All the other properties (*n* = 12) were breeding operations that raised deer for a variety of reasons, including trophy bucks for hunting ranches and animal products such as antlers and urine.

### 2.3. Field Methods

**Figure 1:**
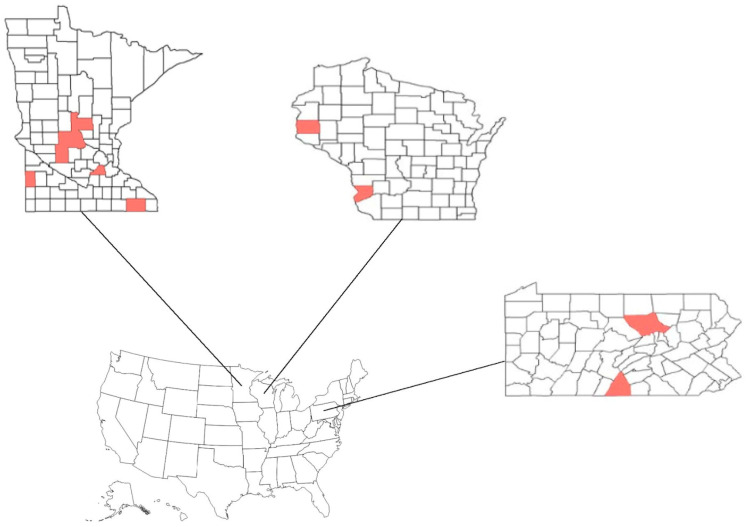
Counties where study farms were located and each state’s relative location in the United States. We name the graphics from the top left clockwise. Minnesota Counties (**top left**): Fillmore, Kandiyohi, Lincoln, Morrison, Scott, Stearns. Wisconsin Counties (**top right**): Crawford, St Croix. Pennsylvania Counties (**bottom**): Franklin, Lycoming. Lines connect each state to its approximate location on the U.S. map to illustrate geographic context.

To quantify wildlife activity on the properties, we placed trail cameras on fences around 14 white-tailed deer (deer; *Odocoileus virginianus*) farms in Minnesota, Wisconsin, and Pennsylvania (Figure 1); in addition, we also placed cameras directed at feeders within 6 of the properties. Properties were monitored during fall, winter, and summer seasons. All properties were monitored during the summer season (*n* = 14; 1 June–17 August). In addition, a portion of properties were monitored during the winter (*n* = 4; 3 February–7 April), and another subset was monitored in the fall (*n* = 2; 11 October–30 November). Properties were monitored for at least 14 days per season; during this time, all focal species (i.e., bobcat (*Lynx rufus*), coyote (*Canis latrans*), red fox (*Vulpes vulpes*), Virginia opossum (*Didelphis virginiana*), raccoon (*Procyon lotor*), deer, striped skunk (*Mephitis mephitis*), black bear (*Ursus americanus*), and unidentified mustelid species (*Mustelid* spp.) were identified.

### 2.4. Camera Placement

Properties were monitored using Bushnell 32MP CORE DS-4K No Glow Trail Cameras. Cameras were set to take images on movement and at regular (5 min) intervals using the field scan setting. The motion trigger could not be disabled; however, we set the cool-off period to 30 min to ensure that at most 48 motion-triggered images were captured per day. The image size setting was set to high (32 MP daytime, 8 MP night vision) to aid in identifying smaller animals.

We placed cameras along the fence in a stratified manner according to the proportion of land-cover types available (Table 2). Land-cover types were determined by the 16-level 30 m resolution National Land Cover Database raster [22] (NLCD). The number of cameras placed on a facility varied based on facility size (mean = 13.4, sd = 5.24).

**Figure 2:**
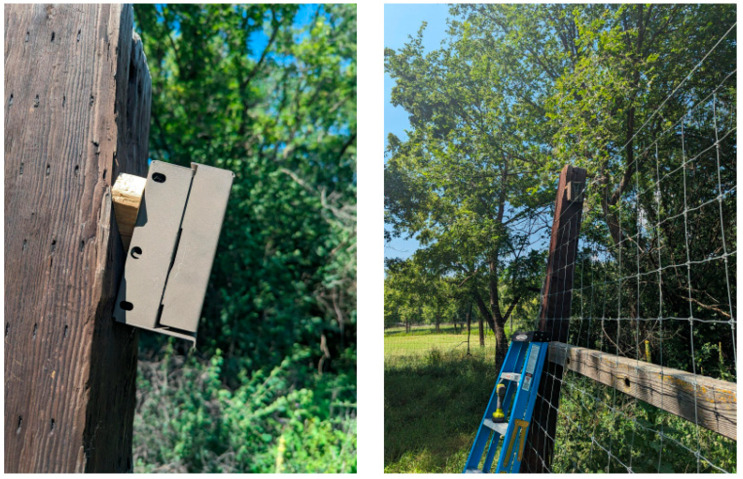
Image of camera placed on fence post displaying camera angle relative to the fence post (**left**). Cameras were placed between 10 and 20 degrees relative to the fence post. Image of camera displaying its view along the fence (**right**).

Cameras were placed in protective metal boxes, and mounted onto fence posts (Figure 2), and were aimed so that the viewshed showed both the inside and outside of the fence. Cameras were installed at a mean height of 259.8 cm (sd = 20.88 cm), and a mean angle of 14.14° (sd = 5.87°). The minimum distance between any two cameras was 30 m (n.b., the maximum distance between cameras varied due to facility size and availability of fence posts; mean = 124.9 m, var = 26,474.2 m). Cameras that were aimed at feeders were attached to fence posts in the same manner as fence cameras; however, these cameras were angled so that they pointed directly at the feeders.

### 2.5. Data Processing

#### 2.5.1. Landscape Metrics

We buffered polygons of each property at 3 spatial scales (i.e., 1, 3, and 5 km) and then subtracted the farm polygons. Within each buffer, we calculated road density using state road layers [23,24,25] and land-cover statistics using the NLCD database [22]. To describe the composition and arrangement of the land-cover within each buffer, we calculated Shannon’s diversity index (diversity of land-cover types; *lsm_l_shdi*) and an aggregation index (a measure of the aggregation of land-cover types; *lsm_l_ai*) from the R-package landscapemetrics [26] (n.b. the 16-class NLCD dataset was used for this). We also calculated the proportion of each land-cover type within the buffers as well as the land-cover at each camera location, but for this we aggregated the 16 NLCD classes into four coarse classes: agricultural, developed, forest, and wetland (coarse land cover). All spatial data were calculated using the sf, terra, and landscapemetrics packages in R [27,28,29].

**Figure 3:**
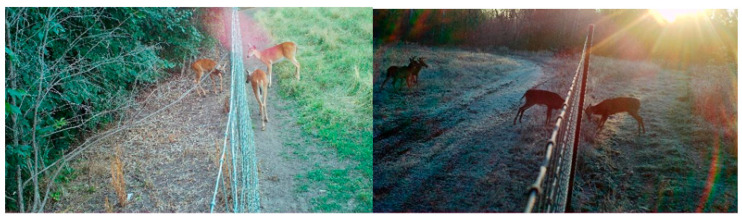
Images where deer were within one body length of one another. An image labeled as “contact possible” (**left**) with wild deer on the left-hand side of the image and farmed deer on the right-hand side. The second image (**right**) was classified as “contact observed”; the wild deer (**right**) is pictured sparring with a farmed deer (lef2.5.2. Megadetector).

The workflow for classifying camera-trap images followed an automated-manual review paradigm [30], utilizing a publicly available image classification model, Megadetector Version 5 [26] (MDv5). The MDv5 model was used to differentiate images that likely contained animals (positive) from those that likely did not (negative) within some user-defined confidence threshold; this threshold was a measure of the algorithm’s confidence in its classification. A confidence threshold of 0.25 would instruct Megadetector to classify anything with a 25% certainty or greater to be a positive image. This 0.25 confidence threshold was suggested in the literature [31]; however, we adopted an even more conservative threshold of 0.1 to reduce the chance of Megadetector missing an animal in the frame. After classification, Megadetector generated a JSON file which contained its categorization of an image set along with its confidence in that categorization. Images denoted as positive were classified exhaustively by a trained technician (the technician noted the type and number of each species observed and their location relative to the fence). Images that contained wild and farmed deer within one body length of one another on either side of the fence were further classified based on type of contact (Figure 3). Lastly, two hundred negative images from each camera were randomly sampled again by the technician as a quality control check for false negatives.

#### 2.5.2. Independent Observations

To reduce the probability of counting the same individual more than once, a cutoff time interval (i.e., 30 min) was established to determine “independence” of observations [32]. That is, two or more observations were considered independent observations (hereafter observations) if they were (1) of two distinct species either within or outside of the 30 min interval or (2) of the same species outside of the 30 min interval. Observations at different cameras were assumed to be independent, regardless of timing. On occasion, individuals remained stationary within the viewshed for longer than 30 min and these were not counted as separate observations. This was generally inferred by the arrangement of individuals. No attempt was made to uniquely identify individuals.

### 2.6. Statistical Analysis

Summary statistics of wildlife observations over seasons were derived by first filtering those data into two-week time frames. For instance, for each camera deployment we filtered out any images which were taken outside of the two-week sampling window. Then observations of each class (i.e., scavengers or wild deer) were summed across a given season and the denominator was the total number of images taken in a given season in the two-week sampling window. Summary statistics were computed in similar ways to derive descriptions of observations at feeders as well as placement relative to the fence.

Due to the small sample size of properties observed in winter and fall, we performed inferential statistical analyses on the subset of observations from fence cameras during the summer sampling season only. To analyze how site characteristics affected the number of deer and scavengers observed at each fence camera and farm, we fit two sets of mixed-effects negative binomial models (one set for the number of wild deer observed and another for the number of scavengers observed) using *glm.nb* from the *lme4 R* package (version 1.1.35.3) [33] that account for overdispersion in the data.

Using the count at each camera as the response variable and the farm as a random intercept (the base model), we considered a suite of models. The same set of models was applied to both the wild deer and scavenger groups. Models were fit by adding individual covariates to the base model. The covariates tested included the following: camera height, the number of cameras on each site (camera number), camera angle, the number of hours a camera was functional (number of active hours), the aggregation of different land-cover classes, the proportion of landscape types (i.e., agriculture, developed, forest) in differing buffer sizes, differences in land-cover proportions between different spatial scales [34] (e.g., agriculture 3 km versus 1 km difference), the landscape type at each camera according to the coarse land-cover classes (i.e., camera level), road density at differing buffer sizes, and the number of deer or scavengers observed at a camera (for the opposite group). The top model for each group (wild deer and scavengers) was selected by minimizing Akaike’s Information Criteria [35] (AIC).

## 3. Results

### 3.1. Wildlife at Farm Fences

We captured 697,450 images in the summer, 220,219 images in the fall, and 273,135 in the winter along fences. This resulted in 749 observations of focal species. Of those observations, 77% (*n* = 574) were wild deer, 22% (*n* = 164) were scavengers, and an additional 1% (*n* = 10) were not identifiable. Wild deer were observed exclusively outside of the fence. The average number of observations of wild deer across all sites during the summer was 12.74 over the two-week observation period.

Scavengers were observed both inside and outside the fence in almost equal measure. In total, 43% (*n* = 71) of scavenger observations were inside of the fence, 54% (*n* = 88) were observed outside of the fence, and an additional 3% (*n* = 5) were observed on the fence itself. Of the observations of scavengers, 60.6% (*n* = 100) were raccoons, with the remaining 39% composed of seven other species (Table 3). Coyotes were the second-most frequently observed scavenger (*n* = 26), but only one was observed inside of a fence. The remaining scavenger species were infrequently observed, and those observations were nearly evenly distributed outside and inside the fence; however, some species were mostly observed inside (bobcats, bears, and a mustelid) and some were mostly observed outside (foxes, opossum, skunk). We observed an average of 9.20 scavengers per week across all sites.

### 3.2. Wildlife at Farmed Deer Feeders

**Figure 4:**
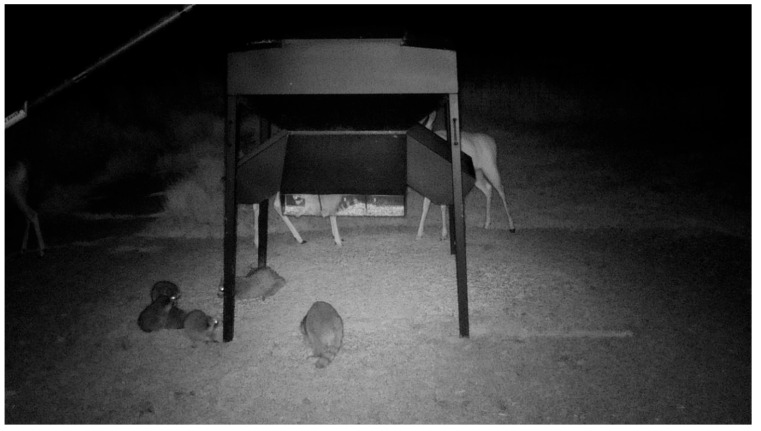
Raccoons sharing a feeder with a farmed white-tailed deer. A raccoon is visible inside of the feeder, a common occurrence observed on the farms.

We made 161 observations of scavengers along fences from 1.13 M images taken. By comparison, we recorded 130 observations of scavengers from 57,637 feeder images (16 times greater than fences; Figure 4). Fence line cameras recorded an average of 0.29 observations per week while feeder cameras recorded 4.41 observations per week.

Group sizes of scavengers at feeders were larger than group sizes of scavengers observed along fences (mean = 1.5, sd = 1.05). Group sizes of scavengers would sometimes reach up to eight individuals (mean = 1.79, sd = 0.93). These groups frequently congregated for hours at a time or sometimes entire nights. Although group sizes along the fences varied more, group sizes at feeders were consistently higher. The mean number of minutes elapsed during an observation of a scavenger at a feeder was between 103.61 min and 108.61 min (sd = 47.5 min).

### 3.3. Wild and Farmed Deer Interaction

We captured 574 observations of wild deer; only 45 (~8%) contained wild and farmed deer within one body length of one another. Only 5 observations (~1%) showed wild and farmed deer contacting each other through the fence (Table 4). All the direct contacts occurred in the fall despite this season comprising only 20% of our image total. The amount of time elapsed during an interaction between wild and farmed deer was between 6.66 and 11.66 min (n.b., this range is due to the five-minute interval between field scan images). The median number of wild deer at the fence during these interactions was 1 (max = 2). A variety of behaviors were observed in the “contact observed” category including sparring, parallel walking, rutting, and rubbing against bordering trees or fence posts.

### 3.4. Assessment of Association with Landscape Factors

Variables identified with the number of wild deer observations in the top model included the following: the base model, the number of camera active hours, and the coarse land–cover classification (Table 5). Fewer wild deer were observed at cameras surrounded by developed land cover. The number of observations of wild deer and the number of hours that a camera was active was positively correlated with the odds of observing a wild deer.

Fewer variables were associated with scavengers than wild deer. Variables identified with the number of scavenger observations in the top model included the following: the base model and the number of deer observed at the camera (Table 6).

## 4. Discussion

We utilized a network of cameras to test the a priori hypothesis that we expected to observe more wildlife interactions on farms surrounded by more natural habitat and that interactions between wild and farmed deer might vary by season. In light of recent publications demonstrating the ability of scavengers to pass infectious prions through their alimentary tract [14,15,16], our study highlights the fact that transmission of CWD into deer farms might include seasonally mediated behaviors and more complex transmission involving multiple animal species.

Although research surrounding causes of CWD introduction into deer farms has focused on interactions between wild and farmed deer, our results indicate that these interactions through the fence were very limited in their scope and frequency. The few direct contacts through the fence line that were observed occurred in the fall between males that were displaying agonistic behaviors, suggesting that CWD transmission, if it were to occur, is more likely in the fall.

While interactions between wild and farmed deer were very rare, and occurred only through the fence, interactions between farmed deer and scavengers were more likely, as these animals readily crossed over and through the fence to enter deer pens. Raccoons were most frequently observed. Several scavengers have been documented as CWD fecal shedders [14,15,16], though published studies in raccoons are limited to date [15].

The data we collected at feeders combined with lack of associations detected between scavenger observations at perimeter fences with landscape variables evaluated in this study (Table 5 and Table 6) suggest that farming practices (and more specifically deer feeding practices) may be a major driver of scavenger incursions into deer farms. Although the possibility remains that we were unable to detect an effect of landscape on observations of scavenger visitation for logistical (or other) reasons, we posit that results from our statistical analysis (Table 5 and Table 6) further suggest that farming practices (e.g., feeding) may drive scavenger-farmed deer interactions on farms, and not landscape factors as previously hypothesized. Evidence supporting this claim is that cameras placed by feeders had much higher rates of observations of wild scavengers than cameras placed along fences. These conclusions underscore that scavengers at feeders may pose an understudied epidemiological risk to farmed deer, particularly because scavengers tended to stay for long periods of time at feeders (approximated mean = 105 min), providing the opportunity to defecate in farmed deer feeders. If these scavengers had previously consumed tissues of infected wild deer surrounding the properties, they could serve as a vector for CWD transmission through fecal contamination of farmed deer feeds [9]. Further, our wild deer model results indicated that observations of wild deer near deer farm perimeter fences were more likely in areas with less developed land cover, indicating the potential for higher risk of indirect transmission of CWD from wild deer through scavengers to deer farms in these areas (though this was not evaluated in this study). This transmission pathway may be very important in areas of high CWD+ prevalence in wild deer, where hunter harvest, predation events, or other mortality of infected cervids could be consumed by scavengers—which are known to be regular visitors at deer offal sites [36].

Our study was limited by several factors including small sample size and data loss. Due to the political sensitivity of CWD regulations on deer farms, many producers were unwilling to participate in this study, due in part to fear of increased regulations that have already forced many producers to leave the business. In terms of data loss, 48 of the 111 trail cameras in this study experienced some form of data loss or data corruption. The most frequent source of data loss came from our cameras shutting off prematurely. We further reduced our data by restricting our analysis to independent observations at least 30 min apart which some researchers consider unnecessary [37,38,39]. We also found it difficult to ascertain when direct contact occurred between wild and farmed deer because we minimized the number of infra-red triggered images in favor of a 5 min field scan approach (we made this decision so that our data cards were not prematurely filled with images of farmed deer). Additionally, the two-week time period that we utilized for this study may have limited our assessment of wildlife densities in our study areas; 3 or even 4 weeks may be preferable [40]. Lastly, we were not able to collect data from all properties in fall (*n* = 2) and winter (*n* = 4). This is important, as many animals (including deer) change behavior seasonally and these behavioral changes (e.g., rutting) likely impact changes in potential vectors.

Future ecological studies of cervid farms would provide insight into conspecific and interspecific interactions that may drive CWD spread. In conjunction with camera traps, future studies could directly evaluate CWD transmission through linking specific behaviors and farming practices with CWD environmental testing using tools such as RT-QuIC and eDNA [41]. Further, long-range dispersal of CWD through avian scavengers, not yet well assessed, could provide insights into additional ecological mechanisms that drive spread [12,16].

This information will aid deer farmers as they adjust their biosecurity practices to reduce the probability of CWD incursion into their properties. In addition, developing a better understanding of how CWD spreads in the landscape will help wildlife management professionals refine their management of wild cervid herds. Ultimately, CWD transmission is a multifaceted issue involving many aspects of ecology, cervid management, and government regulation. Future studies of farmed cervid facilities should focus on the risks posed by mammalian scavengers and how different farm management practices can improve overall farm biosecurity.

## Figures and Tables

**Table 1:** Description of sites, the states they were sampled from (Minnesota, Wisconsin, Pennsylvania), their size in square km, the type of operation (breeding (B) or hunt ranch (HR)), as well as which season they were sampled where “S” denotes summer, “F” denotes fall, and “W” denotes winter. Some sites were sampled for multiple seasons and those seasons are separated by commas.

Site	State	Size (Sq. km)	Type	Season
1	MN	0.078	B	S, W
2	MN	0.028	B	S, F, W
3	MN	0.018	B	S, W
4	WI	0.478	HR	S, F, W
5	MN	0.004	B	S
6	MN	0.021	B	S
7	MN	0.041	B	S
8	MN	0.005	B	S
9	MN	0.069	B	S
10	PA	8.328	HR	S
11	WI	0.058	B	S
12	PA	0.072	B	S
13	MN	0.051	B	S
14	MN	0.007	B	S

**Table 2:** Column 1 describes the land-cover type and column 2 describes the number of cameras placed in the cover type from the reduced land-cover types described from the NLCD-19 dataset.

Land-Cover Type	Number of Cameras
Agriculture	38
Developed	16
Forest	64
Grassland	35
Wetland	10

**Table 3:** Observations by species and location observed relative to the fence. The numeric columns represent the total number of observations relative to the fence (inside, outside, and on the fence), followed by the proportion (p) of the total observations for that species that each count represents.

Species	Number of Observations Inside the Fence (p)	Number of Observations Outside the Fence (p)	Number of Observations on the Fence (p)
black bear	5 (1.00)	0 (0.00)	0 (0.00)
bobcat	4 (0.8)	1 (0.20)	0 (0.00)
coyote	1 (0.04)	25 (0.96)	0 (0.00)
*Mustelid* spp.	1 (1.00)	0 (0.00)	0 (0.00)
red fox	2 (0.29)	5 (0.71)	0 (0.00)
opossum	2 (1.00)	0 (0.00)	0 (0.00)
raccoon	50 (0.50)	46 (0.46)	4 (0.04)
skunk	2 (0.29)	5 (0.71)	0 (0.00)
spp. unknown	11 (0.52)	9 (0.43)	1 (0.05)
white-tailed deer	0 (0.00)	574 (1.00)	0 (0.00)

**Table 4:** The number of wild WTD IOs by season, site, and type of contact (described in methods). These rates were converted to a weekly expected rate by multiplying the hourly rate (i.e., the total number of camera hours on a deployment) by the number of hours in a week (168).

Site	Season	Number of Observations	Number of Observed Contacts
1	F	0.26	0.03
1	S	0.04	0.00
1	W	0.14	0.00
2	F	0.20	0.05
2	S	0.00	0.00
2	W	0.08	0.00

**Table 5:** Best model for explaining the number of wild white-tailed deer observed at a camera. Developed represents the effect of cameras located within a developed land-cover type. Number of Hours is the amount of time a camera was active.

Predictors	Log Mean	Std. Error	CI	*p*
Intercept	0.31	0.26	−0.20–0.82	0.237
Independent observations of scavengers	0.16	0.10	−0.04–0.35	0.115
Developed land cover	−1.36	0.66	−2.64–−0.07	0.039
Number of hours	0.22	0.16	0.04–0.65	0.025
**Random effects**				
Sigma-squared	1.35			
Tau	0.38			
ICC	0.22			
N	14			
Observations	163			
Marginal R2/Conditional R2	0.170/0.352			

**Table 6:** Best model explaining the number of scavengers observed at each camera.

Predictors	Log Mean	Std. Error	CI	*p*
Intercept	−1.16	0.36	−1.87–−0.82	0.001
Independent observations of deer	0.31	0.14	0.04–0.58	0.024
**Random effects**				
Sigma-squared	1.63			
Tau	1.12			
ICC	0.41			
N	14			
Observations	163			
Marginal R2/Conditional R2	0.034/0.428			

## Data Availability

The data sets presented in this article are not readily available due to privacy concerns involving study participants. Requests to access the datasets should be directed to alex.jack5507@gmail.com.
